# Supplementary figures and images for: Macrophages regulate gastrointestinal motility through complement component 1q
Source: eLife. 2023 Apr 26;12:e78558. doi: 10.7554/eLife.78558 (PMC10185340; doi:10.7554/eLife.78558)

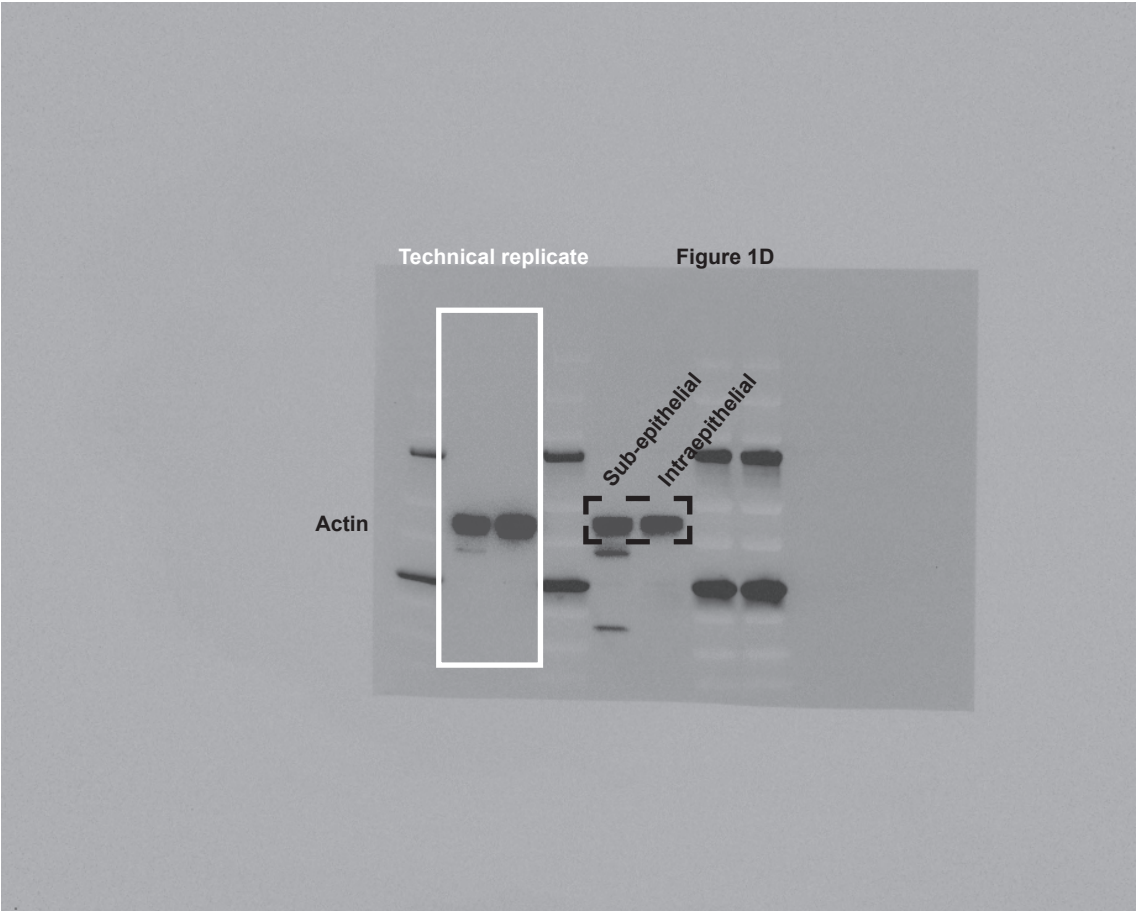

Supplement: Figure 1—source data 1. [file elife-78558-fig1-data1.zip › Figure 1 - source data 1/Figure 1D - source data 1 - Actin - annotated.pdf]

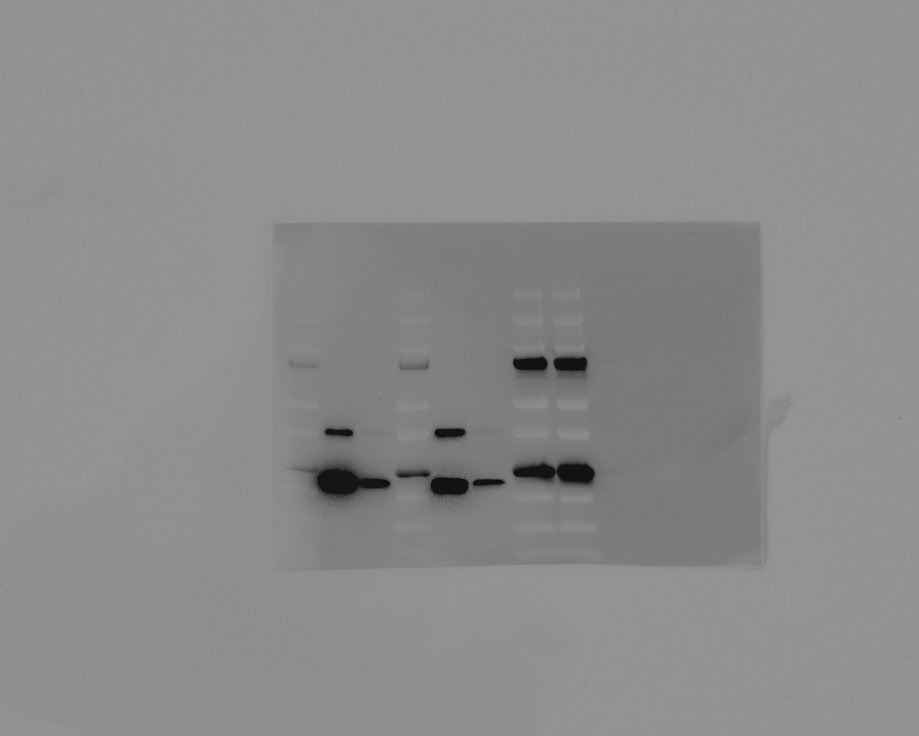

Supplement: Figure 1—source data 1. [file elife-78558-fig1-data1.zip › Figure 1 - source data 1/Figure 1D - source data 1 - C1q.tif]

Technical replicate

Figure 1D

C1q

Sub-epithelial  
Intraepithelial

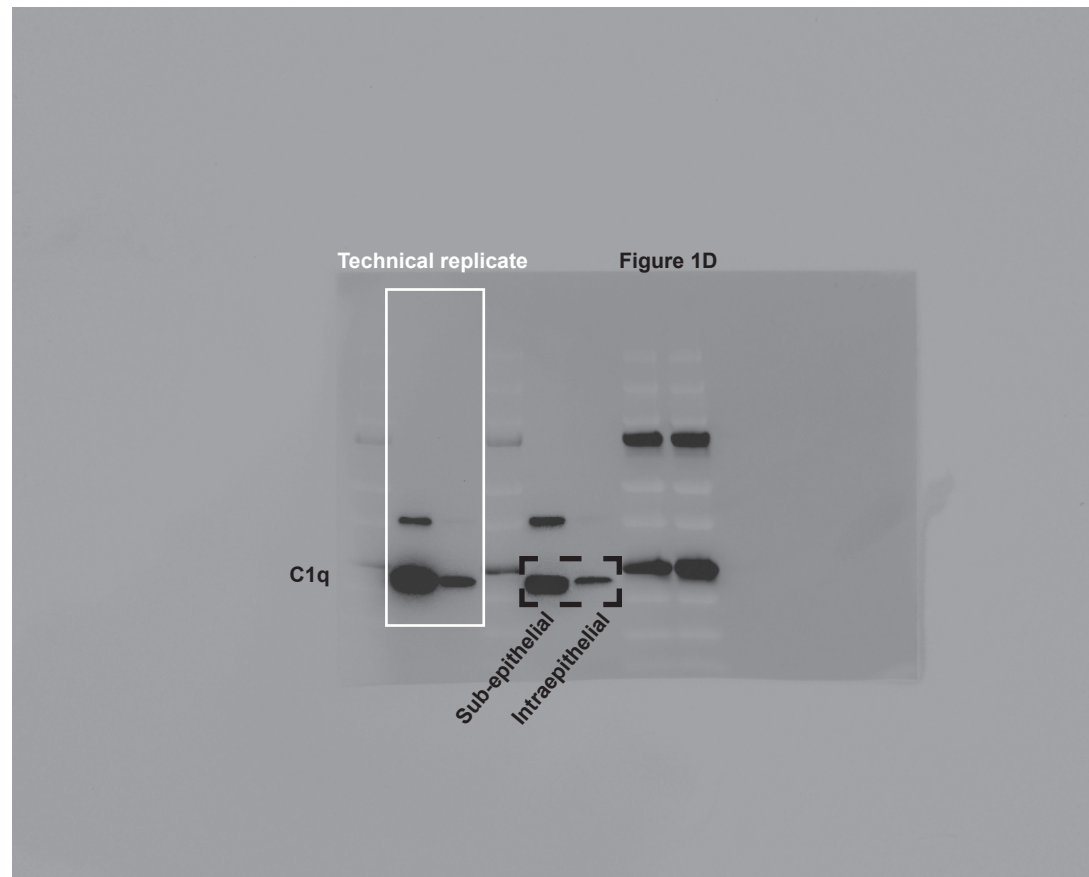

Supplement: Figure 1—source data 1. [file elife-78558-fig1-data1.zip › Figure 1 - source data 1/Figure 1D - source data 1 - C1q - annotated.pdf]

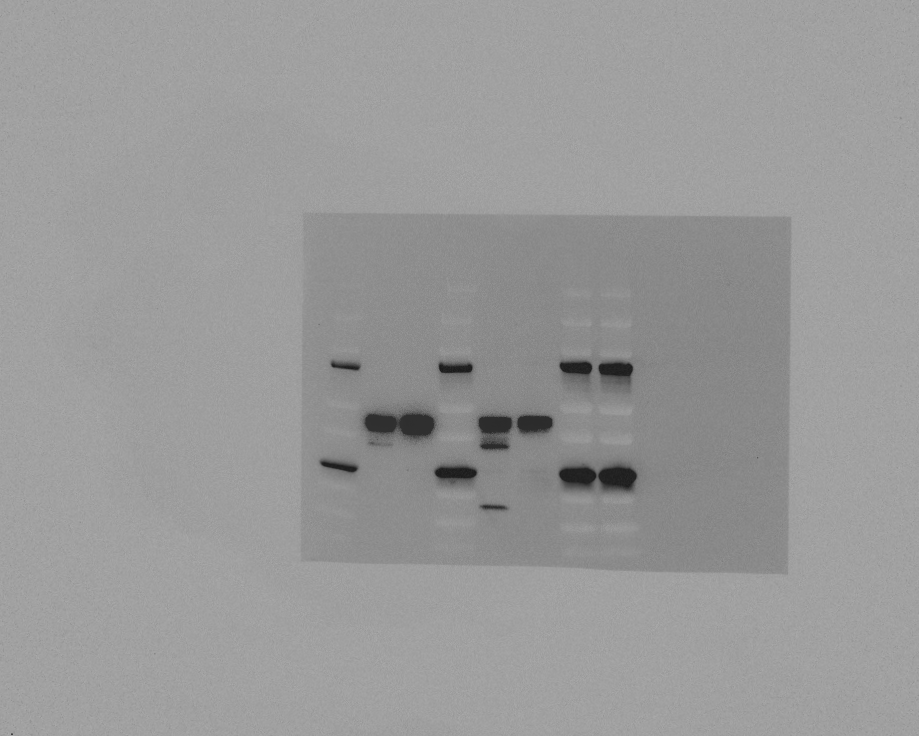

Supplement: Figure 1—source data 1. [file elife-78558-fig1-data1.zip › Figure 1 - source data 1/Figure 1D - source data 1 - Actin.tif]

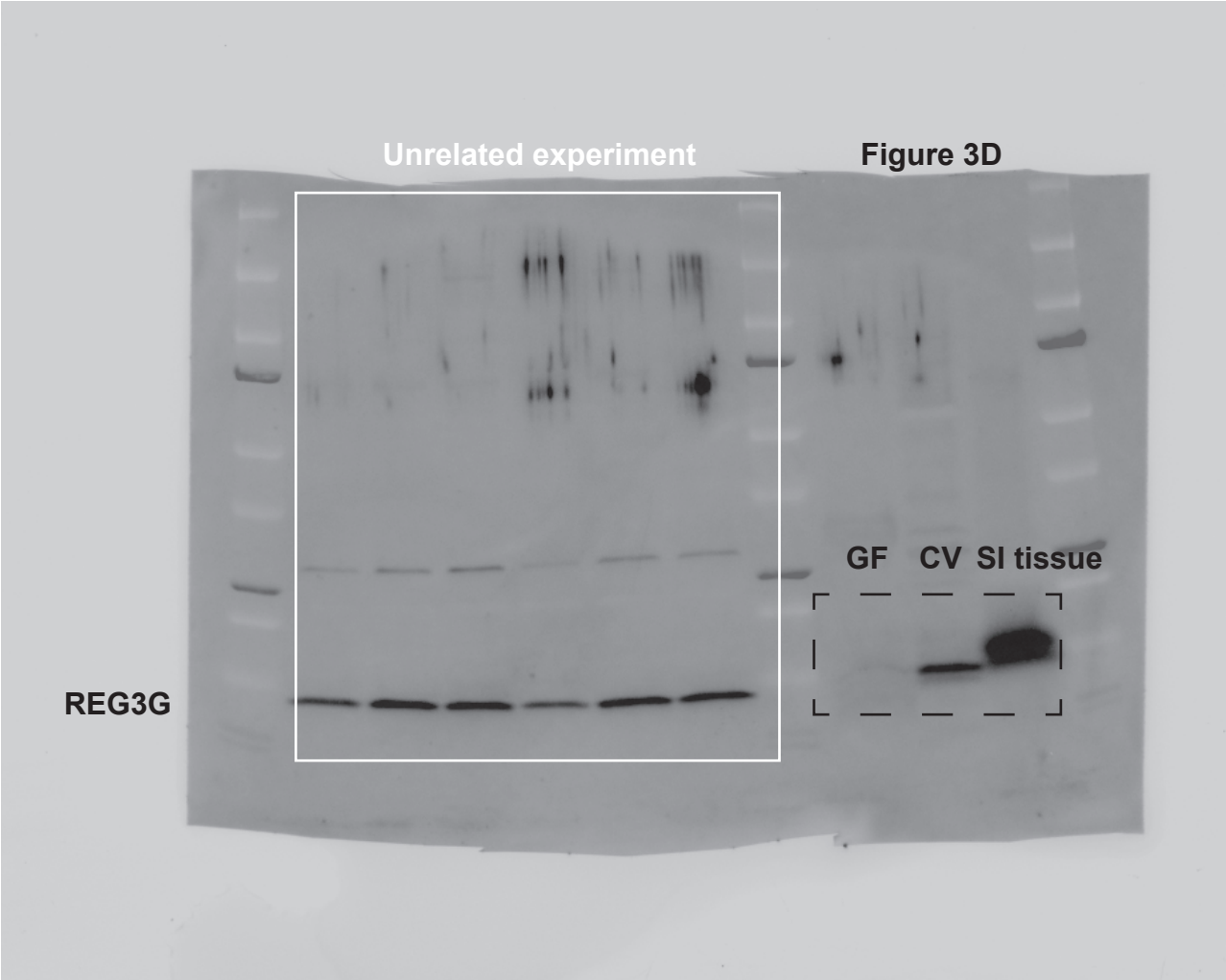

Supplement: Figure 3—source data 1. [file elife-78558-fig3-data1.zip › Figure 3 - source data 1/Figure 3B - source data 1 - Reg3g - annotated.pdf]

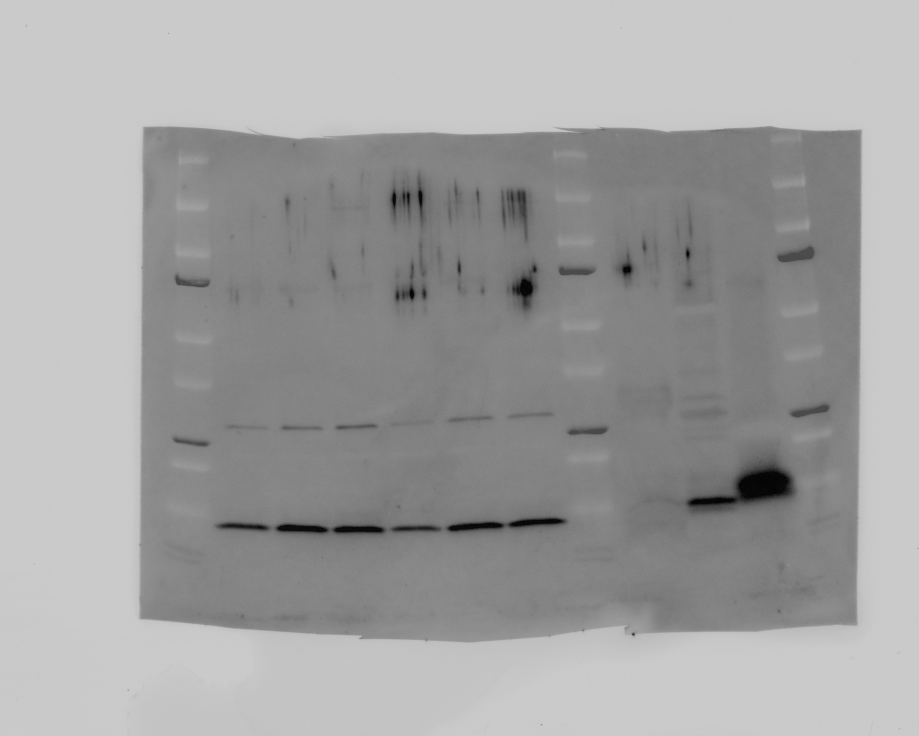

Supplement: Figure 3—source data 1. [file elife-78558-fig3-data1.zip › Figure 3 - source data 1/Figure 3B - source data 1 - Reg3g.tif]

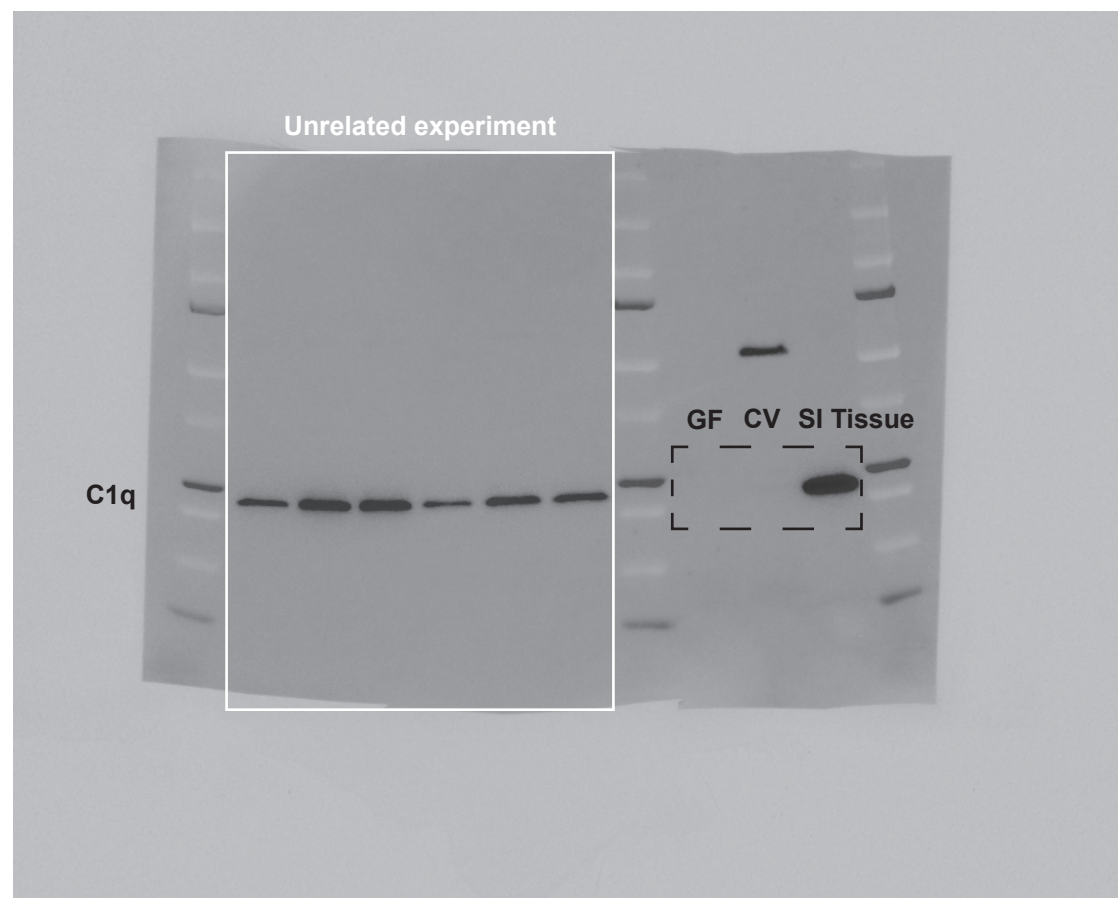

Supplement: Figure 3—source data 1. [file elife-78558-fig3-data1.zip › Figure 3 - source data 1/Figure 3B - source data 1 - C1q - annotated.pdf]

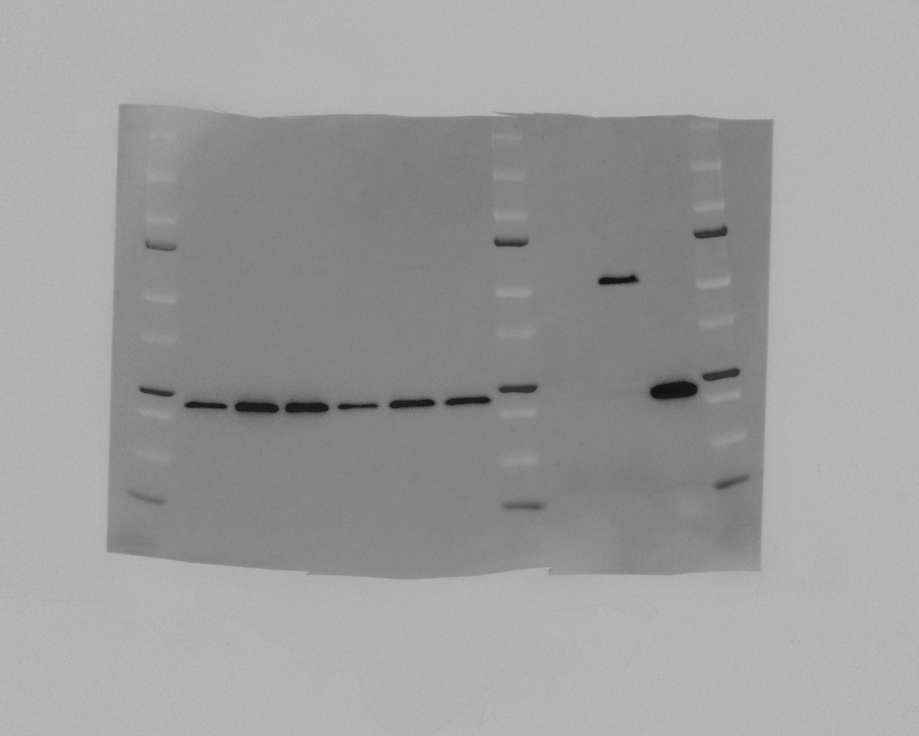

Supplement: Figure 3—source data 1. [file elife-78558-fig3-data1.zip › Figure 3 - source data 1/Figure 3B - source data 1 - C1q.tif]
